# The (Fab)ulous Destiny of Idarucizumab: Highlighting Its Interference with Urine Protein Immunofixation

**DOI:** 10.1055/s-0039-1697642

**Published:** 2019-09-16

**Authors:** Nicolas Gendron, Héloïse Flament, Elena Litvinova, Sofia Ortuno, Nadine Ajzenberg, Dorothée Faille

**Affiliations:** 1 Laboratoire d'Hématologie, Hôpital Bichat-Claude Bernard, AP-HP, Paris, France; 2 Université de Paris, INSERM, U1148, Paris, France; 3 Laboratoire d'Immunologie-Hématologie, Hôpital Bichat-Claude Bernard, AP-HP, Paris, France; 4 Université de Paris, INSERM, U1149, Paris, France; 5 Service de Néphrologie, Hôpital Bichat-Claude Bernard, AP-HP, Paris, France

**Keywords:** idarucizumab, dabigatran, reversal, immunofixation, paraprotein

## Abstract

Idarucizumab is a humanized antigen binding fragment (Fab) of a recombinant anti-dabigatran monoclonal antibody (IgG1-kappa) that allows rapid and sustained reversal of dabigatran-induced anticoagulation in case of bleeding or urgent surgery. Herein, we report a very unusual case of dabigatran reversal by idarucizumab in a 79-year-old woman with acute kidney failure admitted to a hospital in a context of hemoptysis. Three repeated injections were necessary because of massive dabigatran overdose and high rebounds of dabigatran plasma concentration. Idarucizumab was found on urine immunofixation up to 6 days after the last injection where it reacted with anti-kappa light chain antibody, but not with anti-gamma heavy chain antibody. Physicians should be aware of the increased half-life of idarucizumab in this context of acute kidney impairment and of its interference with urine immunofixation because it could lead to false-positive results and misdiagnosis of a paraprotein.

## Introduction

Idarucizumab is a humanized monoclonal antigen binding fragment (Fab G1-kappa) that specifically neutralizes anticoagulant effect of dabigatran 1 2 and can be used in emergency situations. 3

**Fig. 1:**
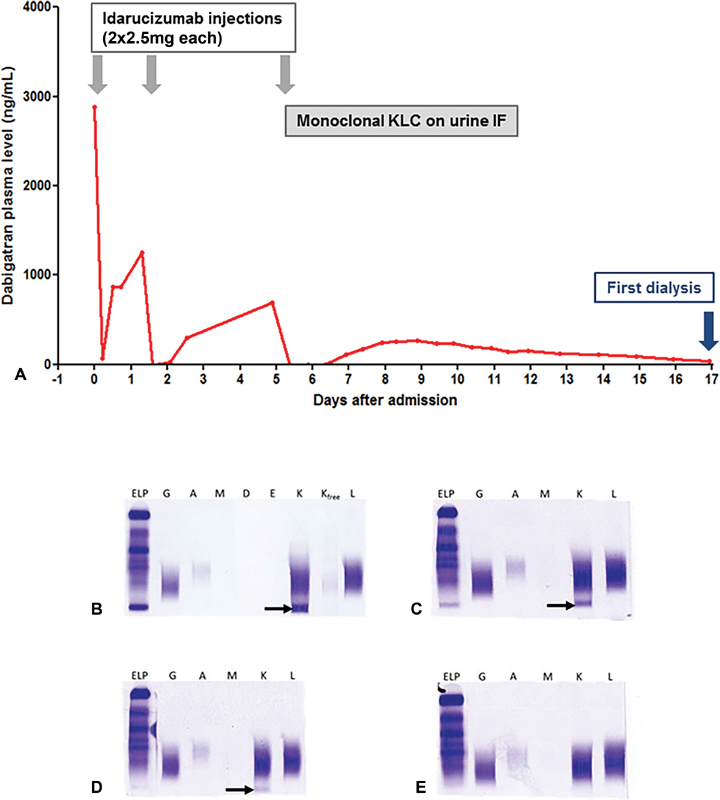
Plasma dabigatran concentration ( **A** ) and urine immunofixation ( **B–E** ) after three injections of idarucizumab. At day 5 (B), monoclonal kappa light chains (KLC, *black arrow* ) with no corresponding gamma, alpha, delta, or epsilon heavy chains and no corresponding free KLC. Progressive disappearance of KLC at days 9 (C) and 11 (D). Complete disappearance at day 12 (E).

A 79-year-old woman was admitted to an emergency department for hemoptysis and progressive asthenia. She was taking dabigatran etexilate (150 mg twice daily) for nonvalvular atrial fibrillation. On admission, her full blood count revealed a hemoglobin concentration of 80 g/L, mild leucocytosis, and a normal platelet count. Laboratory investigations showed elevated urea and creatinine levels at 40.9 mmol/L and 1,031 μmol/L respectively, associated with massive dabigatran overdose (plasma concentration 2,881 ng/mL, Hemoclot Thrombin Inhibitor, Hyphen BioMed). She received two first intravenous infusions of 2.5 g idarucizumab within 15 minutes of each other few hours after admission. Two additional injections (2 × 2.5 g each) were performed at days 2 and 5 due to high rebound of plasma dabigatran after each reversion ( Fig. 1A ) and because a kidney biopsy was then considered, although no bleeding event was observed during hospitalization. Serum and urine immunofixation (IF) were performed to exclude multiple myeloma in this context of isolated, nonregenerative normocytic anemia and renal failure. At day 5, serum IF was normal whereas urine IF showed isolated monoclonal kappa light chains (KLCs, Fig. 1B , black arrow). Surprisingly, no free KLC was detected ( Fig. 1B ) and serum free light chain ratio was normal. Urine IF was repeated daily until progressive disappearance of monoclonal KLC ( Fig. 1C–E ). Bone marrow aspiration excluded hematologic malignancies.

The patient was diagnosed with end-stage renal disease and referred to a dialysis center. Anticoagulation was resumed with warfarin. Her last creatinine clearance estimation performed 14 months ago was of 38.5 mL/min (Cockcroft-Gault) and confirmed chronic impaired kidney function. This underlines the importance of regular assessment of renal function in patients receiving dabigatran and of a dose reduction according to the label recommendation in the case of renal impairment.

Idarucizumab, dabigatran, and idarucizumab–dabigatran complexes are mainly excreted in urine. 4 Thus, idarucizumab can be detected on urine IF where it reacts with anti-KLC antibody, but not with anti-gamma heavy chain antibody because it lacks the two heavy chains composing Fc portion. Idarucizumab clearance is reduced in the case of renal impairment, leading to increased half-life and sustained urine excretion. However, one single injection of 5 g idarucizumab was insufficient to neutralize dabigatran. This case also illustrates our previous findings that an initial dabigatran plasma level ≥200 ng/mL before idarucizumab injection could predict dabigatran plasma rebound. 5

In this rare context of acute kidney impairment and repeated injections of idarucizumab, idarucizumab should not be misdiagnosed with paraprotein on urine IF.
